# Two Non-target Recessive Genes Confer Resistance to the Anti-Oomycete Microtubule Inhibitor Zoxamide in *Phytophthora capsici*

**DOI:** 10.1371/journal.pone.0089336

**Published:** 2014-02-20

**Authors:** Yang Bi, Lei Chen, Meng Cai, Shusheng Zhu, Zhili Pang, Xili Liu

**Affiliations:** 1 Department of Plant Pathology, China Agricultural University, Beijing, China; 2 Institute of Plant Protection, Chinese Academy of Agricultural Sciences, Beijing, China; 3 College of Forestry, Beijing Forestry University, Beijing, China; 4 Key Laboratory of Agro-Biodiversity and Pest Management of Education Ministry of China, Yunnan Agricultural University, Kunming, Yunnan, China; University of Nebraska-Lincoln, United States of America

## Abstract

This study characterized isolates of *P. capsici* that had developed a novel mechanism of resistance to zoxamide, which altered the minimum inhibition concentration (MIC) but not the EC_50_. Molecular analysis revealed that the β-tubulin gene of the resistant isolates contained no mutations and was expressed at the same level as in zoxamide-sensitive isolates. This suggested that *P. capsici* had developed a novel non-target-site-based resistance to zoxamide. Analysis of the segregation ratio of zoxamide-resistance in the sexual progeny of the sensitive isolates PCAS1 and PCAS2 indicated that the resistance to zoxamide was controlled by one or more recessive nuclear genes. Furthermore, the segregation of resistance in the F_1_, F_2_, and BC_1_ progeny was in accordance with the theoretical ratios of the χ^2^ test (*P*>0.05), which suggested that the resistance to zoxamide was controlled by two recessive genes, and that resistance to zoxamide occurred when at least one pair of these alleles was homozygous. This implies that the risk of zoxamide-resistance in *P. capsici* is low to moderate. Nevertheless this potential for resistance should be monitored closely, especially if two compatible mating types co-exist in the same field.

## Introduction

*Phytophthora capsici* Leonian is a devastating oomycete plant pathogen with a global distribution [1]–[4]. Since it was first isolated in New Mexico in 1918, *P. capsici* has been found to cause severe epidemics in a wide range of Cucurbitaceae and Solanaceae hosts [5]. And more recently, it is also known to affect tropical crops, leguminous plants, the coniferous tree Fraser fir (*Abies fraseri* (Pursh) Pior.) and several weed species [2], [6]–[9]. *P. capsici* is a heterothallic, diploid oomycete, which reproduces sexually when A1 and A2 mating types interact [2]. The thick-walled oospores produced contain novel gene combinations and can survive in the soil for years [2], [10]. Consequently, *P. capsici* populations often have a high level of diversity and can rapidly adapt to new conditions by a loss of heterozygosity (LOH) [11]. The main method of control for *P. capsici* has been the application of fungicides in combination with resistant cultivars and cultural practices [12], [13]. However, the emergence of fungicide resistance and adaptation to novel hosts in recent years has raised the profile of this devastating disease [6]–[9], [14], [15].

Several fungicides groups with diverse modes of action are currently available for the control of oomycete diseases including: phenylamides (e.g. metalaxyl), QoI inhibitors and carboxylic acid amides (CAA) [16]–[18]. However, populations resistant to these fungicides have emerged in many oomycete pathogens [18], [19]. The evolution of resistance in these species is driven by multiple factors including mutation, sexual reproduction, recombination and natural selection. However, the availability and rate of mutations conferring resistance and their associated genetic mechanisms and fitness costs all have the potential to effect the evolution of resistance in these pathogens [20]. Research investigating the mechanism of resistance to phenylamides has found that one (or two) major gene (s), and potentially several minor genes can contribute to resistance in different oomycete pathogens [21]–[25]. This genetic background has led to the rapid development of resistance to metalaxyl in many oomycetes [19]. In contrast, resistance to QoIs has been linked to amino acid substitutions in the cytochrome b protein, which is controlled by a maternal gene, indicating that the evolution of resistance to QoIs depends mainly on mitosis [26]. Meanwhile, resistance to CAA fungicides in *Plasmopara viticola* has been found to be controlled by one recessive mutation in the cellulose synthase 3 (CESA3) [27], while resistance to flumorph in *P. capsici* is controlled by two dominant genes [28]. Understanding the mechanism of inheritance for fungicide resistance in oomycetes is therefore a significant factor that could help to predict the occurrence of fungicide resistant isolates and limit their spread.

Zoxamide (commercial name: Gavel or Electis) is the only benzamide fungicide that can be used to control oomycete diseases [29]. It disrupts the microtubule cytoskeleton by binding to β-tubulin [29], and results in the reduced production of sporangia, thereby compromising zoospore release [30]. Zoxamide is also effective against several ascomycete fungi including: *Botrytis cinerea*, *Venturia inaequalis*, *Monilinia fructicola*, *Mycosphaerella fijiensis* and *Cercospora beticola* [31]. Since its introduction in 2001, there have been no reports of resistance to zoxamide for plant pathogens in the field [1], [32], [33]. Furthermore, most attempts to induce zoxamide-resistance in laboratory experiments have failed [33], with the exception of several stable zoxamide-resistant isolates of *P. capsici* that were obtained by treating either mycelial cultures or zoospores with UV irradiation and selecting with zoxamide [1]. In contrast, the many benzimidazole (BZ) and *N*-phenylcarbamates (NPC) fungicides, which share a similar mode of action, arresting nuclear division by binding to β-tubulin, have faced serious resistance problems with many fungal pathogens [29], [34]–[37].

Plant pathogens employ many strategies to overcome the toxicity of fungicides and develop resistance, including alterations to the target site; overproduction of the target site; increased efflux or decreased uptake of the fungicide; and metabolic detoxification [35], [38], [39]. For example, many fungal pathogens have developed resistance to BZs via amino acid changes in the β-tubulin protein [35], [39], while the inherent benomyl-resistance of *Colletotrichum acutatum* results from enhanced expression of the β-tubulin 1 gene [40]. However, although mutations in the β-tubulin genes of *B. cinerea* and *C. gloeosporioides* have been linked to zoxamide-sensitivity [32], [41], [42], the mechanism of zoxamide-resistance in oomycete pathogens is still poorly understood.

The current study was initiated to gain greater insight into the zoxamide resistance of *P. capsici*, with three main objectives: to investigate the molecular mechanism of zoxamide resistance; to characterize the segregation pattern of zoxamide-resistance in the sexual progeny; and to assess the fitness of the sexual progeny by investigating their biological characteristics relative to their parental isolates.

## Materials and Methods

### Isolates of *P. capsici*

The isolates used in this study have been listed in Table 1. The sensitive isolates (PCAS1 and HX-1) as well as the resistant mutants (RZ13-2 and HX38-13) have been described previously [1]. To ensure genetic uniformity, the cultures were isolated from single zoospores and maintained on potato dextrose agar (PDA) medium (200 gl^−1^ potato, 18 gl^−1^ glucose, 15 gl^−1^ agar) immersed in mineral oil at 18°C for long-term storage.

**Table 1:** *Phytophthora capsici* isolates used in this study.

Isolate^a^	Origin	Mating type	Sensitivity^b^
PCAS1	California, USA	A1	S
PCAS2	California, USA	A2	S
HX-1	Hebei, China	A1	S
DZ32	Hebei, China	A1	S
SX-2	Shaanxi, China	A2	S
SX-22	Shaanxi, China	A2	S
HN10-1	Anhui, China	A2	S
RZ4-1	Mutant	A1	R
RZ3-5	Mutant	A1	R
RZ13-2	Mutant	A1	R
XH38-10	Mutant	A1	R
XH38-13	Mutant	A1	R

a RZ4-1, RZ3-5, and RZ13-2 are zoxamide-resistant mutants generated from PCAS1 by UV-mutagenesis. XH38-10 and XH38-13 are zoxamide-resistant mutants generated from HX-1 by zoxamide adaption.

b Isolates were only considered resistant (R) when they were able to grow on PDA plates amended with 30 µg ml^−1^ zoxamide.

### Sensitivity Assay

The sensitivity of the *P. capsici* isolates were determined by measuring the mycelial growth on zoxamide (97.5% a.i., Gowan Company, LLC, Yuma, AZ) amended medium according to the protocol of Bi *et al*. [1]. Fresh mycelial plugs (5 mm in diameter) were cut from the edge of actively growing colonies and placed face up on PDA plates amended with a range of zoxamide concentrations: 0, 0.01, 0.02, 0.04, 0.08, 0.1, 0.2, 0.4, 0.8, 1, 2, 4, 8, 10, 20, 40, 80, and 100 µg ml^−1^. After incubation at 25°C in darkness for 6 days, colony growth was measured at perpendicular angles. Each treatment was replicated three times and the experiment conducted at least twice.

### Mating-type Determination

Mycelial plugs of the sample isolates were paired with PCAS1 (A1 mating type) and PCAS2 (A2 mating type) on plates of 10% V8 medium. Mycelial plugs (5 mm) of the progeny were placed in the center of fresh plates with a mycelial plug of PCAS1, PCAS2, and itself to test for self-fertility. The plates were kept in the darkness at 25°C for at least one week before they were examined by light microscopy for the presence of oospores.

### Generation of S_1_-, F_1_-, BC_1_-, and F_2_-populations

All the crosses were performed according to the protocol of Meng *et al*. [28]. Twelve self-crosses and ten crosses were performed in total, using the sensitive and resistant isolates listed in Table 1. In order to obtain adequate quantities of oospore progeny for the segregation of resistance analysis, each self-cross was repeated sixteen times, and each cross four times. Finally, all the oospore progeny from each cross were collected for the subsequent analyses.

The selfing experiments were conducted as follows: parental isolates were first inoculated on plates containing V8 medium and incubated in darkness for 5 days at 25°C. Mycelial plugs (35 mm) were then transferred to the center of 1% water agar plates (mycelium-side up). A 47 mm sterile polycarbonate membrane (Whatman Inc.) was placed on the surface of the plug and a mycelial plug of PCAS2 placed on top (mycelium-side down).

The crossing experiments were conducted using strips (70×30 mm) of different *P. capsici* mating types placed opposite each other on V8 medium plates. The plates were then sealed with Parafilm and incubated in darkness for 14 days at 25°C for oospore production, and then a further 45 days for oospore maturation. The media attached to each side of the membrane as well as the strips of media from the different crossing experiments were ground separately in distilled water using a blender. The homogenized cultures were then vacuum-filtered through a series of sieves with different pore sizes (54 and 31 µm) to remove the media, other residues, mycelia, and sporangia or sporangiophores. The final flow-through aliquot was passed through a 15 µm sieve to collect the oospores, which were washed three times and resuspended in sterile distilled water. The oospores were then treated with a 0.25% KMnO_4_ solution at room temperature for 20 min with shaking at 44×g. Finally, the oospores were collected on a 15 µm sieve and washed with distilled water before being spread (approximately 100 oospores per plate) on S+L medium [43]. The cultures were then incubated for 24–72 hours at 25°C under black light. The resulting single-oospore cultures, representing either the first generation of self-crossing (S_1_) or hybridization (F_1_), were then transferred to PDA and incubated in darkness for 5 days at 25°C.

**Figure 1:**
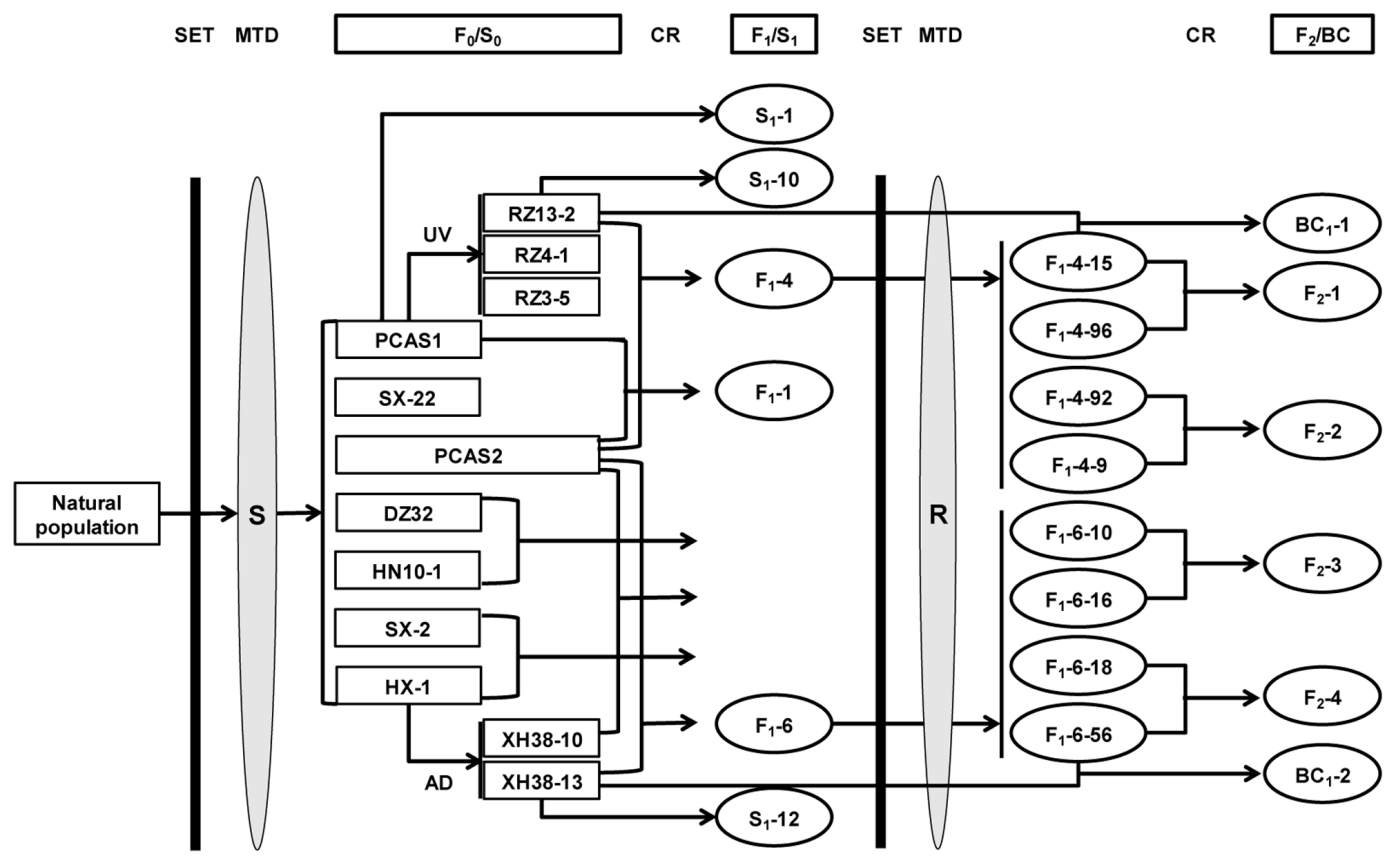
Overview of the crossing strategy used to determine the segregation of zoxamide-resistance in progeny of *P. capsici*. SET, sensitivity test; MTD, mating type determination (A1/A2); OSP, oospore progeny; UV, ultraviolet treatment; AD, adaptation to zoxamide-amended media; CR, cross; SCR, self-cross; S_0_, S_1_, parental isolates and first generation of isolates from self-cross hybridization, respectively; F_0_, F_1_, F_2_, parental isolates and progeny of sexual hybridization, respectively; BC, backcross.

Eight zoxamide resistant F_1_ progeny were then randomly selected for sib-crossing to generate the F_2_ populations. Four zoxamide-resistant F_1_-4 isolates, resulting from the initial crossing of RZ13-2 × PCAS2, were crossed: F_1_-4-15 × F_1_-4-96, and F_1_-4-92 × F_1_-4-9 to produce the F_2_-1 and F_2_-2 populations, respectively (Figure 1). Similarly the F_1_-6 isolates, resulting from the crossing of XH38-13 × PCAS2, were crossed: F_1_-6-10 × F_1_-6-16, and F_1_-6-56 × F_1_-6-18 to produce the F_2_-3 and F_2_-4 populations respectively (Figure 1).

The backcross populations were then produced using the following isolates: F_1_-4-15 × RZ13-2; F_1_-4-92 × RZ13-2; F_1_-6-16 × XH38-13; and F_1_-6-18 × XH38-13, the progeny of which were designated BC_1_-1, BC_1_-2, BC_1_-3, and BC_1_-4, respectively.

### DNA and RNA Extraction and cDNA Synthesis

*P. capsici* isolates were grown on PDA for 4 days at 25°C, and approximately 100 mg of the mycelium scraped from the PDA surface using a sterile pestle. The DNA was then extracted and purified [44] to produce samples suitable for PCR. Total RNA was extracted from mycelial tissue using the RNAiso Plus Kit (Takara Biotechnology Co., Ltd., Dalian, China) according to the manufacturer’s protocol, and cDNA synthesized using the PrimeScript® RT reagent Kit with gDNA Eraser (Takara Biotechnology Co., Ltd., Dalian, China).

### Amplification and Sequencing of β-tubulin Gene

Primer Premier software (version 6.0) was used to create the betaF1/betaR1 primer pair (5′-tccgaattctcctcacagccagc-3′, 5′-ctactcacgaggtgcacgga-3′) based on the sequence of *PcTubB* gene (EF495258) published recently [45]. The PCR was conducted using a 50 µl reaction mixture containing 50 ng of template DNA, 1 µl of each primer (10 µM), 4 µl of dNTP mixture (2.5 mM each dNTP), 1×EasyTaq DNA Polymerase Buffer, and 2.5 U of EasyTaq DNA Polymerase (TransGene Biotech, Beijing, China). The PCR was processed using a MyCycler™ Thermal Cycler (Bio-Rad) with the following program: 5 min at 95°C; 35 cycles of 94°C for 30 s, 62°C for 30 s, and 72°C for 4 min; with a final extension at 72°C for 10 min. The PCR products were separated by electrophoresis using a 1% agarose gel in Tris-acetate (TAE) buffer, purified using the EasyPure PCR Purification Kit (TransGene Biotech, Beijing, China) and sequenced by Beijing Sunbiotech Co. Ltd. (Beijing, China). The DNAMAN (version 7.0) software package was used to predict the amino acid sequences from the DNA sequences and to compare the zoxamide-resistant samples with the wild-type isolates.

### Quantitative RT-PCR Analysis of β-tubulin Gene

Mycelia from the *P. capsici* isolates were used to inoculate a series of 50 ml liquid cultures of PD media amended with zoxamide to produce a range of final concentrations (0, 0.1, 0.5, 1, 5, 10, 50 µg ml^−1^). The cultures were incubated for 6 hours at 25°C in a rotary shaker at 106×g, prior to total RNA isolation and cDNA synthesis (described above).

The real-time PCR was performed using SYBR Green fluorescence and the ABI 7500 System (Applied Biosystems). Fragments of the *PcTubB* gene (108 bp) were amplified with the primer pairs RTtubBF/RTtubBR (5′-ggctatgttccgtcgtaagg-3′, 5′-tactcagacaccagatcgttca-3′), and its expression normalized with the expression of the gene from the 40S ribosomal protein S3A (WS21, GenBank: BT032414), which was amplified using the primer pair RTws21F/RTws21R (5′-ggaaagaacaaacgcctgac-3′, 5′-gttgcgctccgagaagata-3′). The WS21 housekeeping gene was selected because it has previously been shown to be particularly suitable for normalizing expression levels in *P. capsici* [46]. Both negative and positive controls were included in each PCR experiment and all the assays were performed in triplicate using independent PCR runs for each cDNA sample.

The PCR was performed using 20 µl reaction mixtures containing 1×SYBR® Premix Ex Taq™ (Takara), 0.2 µM of each primer, 1×ROX reference Dye II and 2 µl of cDNA template. All the PCRs were processed in triplicate using the following program: 95°C for 30 s, followed by 40 cycles of 95°C for 5 s, 60°C for 34 s and 72°C for 34 s and finally one cycle at 95°C for 1 min and 60°C for 1 min. Melting curve analysis, using 100 cycles increasing in temperature by 0.4°C increments starting at 60°C was performed immediately after amplification to confirm the products had been amplified correctly. The amplification specificity was checked using the resulting melting curves. The 2^−ΔΔCT^ method [47] was used to calculate the expression levels. Two biological replications using fresh cultures for RNA extraction were conducted for each sample, with three technical replicates for each biological replicate.

### Molecular Characterization of Sexual Progeny

Two methods were used to verify the success of the sexual recombination: simple sequence repeat (SSR) and single nucleotide polymorphism (SNP). The SSR used 8 primer sets reported previously [48], which were tested in a preliminary experiment using the parental isolates PCAS2, RZ13-2, and XH38-13 to determine the variability of allele length and to detect null alleles. The forward primers were labeled with fluorescent markers as follows: Pcap1 and Pcap5 were labeled with FAM; Pcap2 and Pcap6 with HEX; Pcap3 and Pcap7 with TAMRA; and Pcap4 and Pcap8 with TET. The PCR was performed using 25 µl reaction mixtures containing 0.1 U of EasyTaq DNA Polymerase (TransGen Biotech, Beijing, China), 1×EasyTaq DNA Polymerase Buffer, 1 µl of dNTP mixture (2.5 mM each dNTP), 0.2 µM forward and reverse primers and 50 ng of DNA template and processed using a MyCycler™ Thermal Cycler (Bio-Rad) and the following PCR program: 95°C for 5 min; followed by 35 cycles of 95°C for 30 s, 60°C for 30 s, 72°C for 45 s with a final extension of 72°C for 10 min. The amplified fragments were separated using an ABI PRISM3100 DNA Analyzer with GENESCAN400HD (ROX) (Applied Biosystems) as the internal size standard at Biomed Co. Ltd. (Beijing, China). The Genemarker® version 1.6 (Softgenetics) software package was used to determine the length of the fluorescently labeled fragments.

The SNP screening utilized fragments of the *CesA3* (GenBank: JX905357) and *CBOT41-P06* (GenBank: BT031614) genes, which were amplified using the following primer sets PCCES3UPPF/PCCES3UPPR (5′-tacgatagcacgggatagg-3′, 5′-cagaaatgaggtcctgtgc-3′) and PCP06PF/PCP06PR (5′-aaggagtgggtgatgaacaa-3′, 5′-tacgatagcacgggatagg-3′), respectively. The *CesA3* gene was used to test the F_1_-4 progeny, while *CBOT41-P06* was used for the F_1_-6 progeny. The PCR was conducted using the same protocol as the SSR analysis but with a lower annealing temperature of 56°C for 30 s. The PCR products were separated, by electrophoresis in a 1% agarose gel in Tris-acetate (TAE) buffer, purified using the EasyPure PCR Purification Kit (TransGene Biotech, Beijing, China) and sequenced by Beijing Sunbiotech Co. Ltd. (Beijing, China). The DNAMAN software package was used to analyze the resulting sequences for SNPs.

### Identification of Zoxamide-resistance in Sexual Progeny

The progeny of the initial sexual crosses were inoculated on plates containing PDA medium amended with 30 µg ml^−1^ zoxamide (PDZ30) [1]. The sensitive and resistant parental isolates were included as controls. PDA plates without fungicide amendment were used as a further control treatment. The cultures were incubated in darkness at 25°C for five to seven days. The surviving colonies were transferred to PDZ30 plates to confirm their resistance. Each experiment was repeated twice.

### Biological Characteristics of F_1_-progeny

#### Temperature tolerance of progeny

Mycelial plugs (5 mm) were used to inoculate PDA plates and the colony diameters measured after incubation in darkness at a range of temperatures; 15°C, 20°C, 25°C, 28°C, and 37°C for 5 days. Each isolate was represented by four replicate plates, and the experiment repeated twice.

Mating type determination of F_1_-progeny. The mating type of the progeny was determined as described above.

#### *In vitro* zoospore production of F_1_-progeny

Mycelial plugs (5 mm) from 5-day-old cultures of the progeny and their parents were placed on carrot agar plates (200 gl^−1^ carrot, 15 gl^−1^ agar). After incubation at 25°C under light (2000 to 3000 lux) for 5 days, 10 ml of sterile water was added to the plates, which were then kept at 4°C for 30 to 45 min to induce zoospore release. The resulting zoospores were counted using a hemacytometer, to determine the number of zoospores per square centimeter of culture. Four replicate plates were used for each isolate, and the experiment repeated twice.

### Virulence of Sexual Progeny

Pepper seedlings (cultivar Tedaqiemen) were grown for six weeks in plastic trays (540×280 mm, 50 seedlings/tray) in a greenhouse. The seedlings were inoculated by adding 2 ml of a 10^4^ zoospore/ml suspension (described above) to the soil surface around the root of plant. The inoculated plants were kept in a greenhouse at 18°C to 25°C and assessed for symptoms of *P. capsici* infection after 9 days, according to a blight severity scale [49]: 0, no visible disease symptoms; 1, leaves slightly wilted with brownish lesions beginning to appear on stems; 2, 30–50% of entire plant diseased; 3, 50–70% of entire plant diseased; 4, 70–90% of entire plant diseased; 5, plant dead. A disease index was then calculated using the following formula: Disease index (%) = 100×(B+2C+3D+4E+5F)/5(A+B+C+D+E+F), where A, B, C, D, E, and F represent the number of plants rated as 0, 1, 2, 3, 4, and 5, respectively. Each experiment was performed twice.

### Statistical Analysis and Genetic Models

The data regarding the biological characteristics of *P. capsici* were analyzed using the general linear model (GLM) function of the Statistical Analysis System (version 9) software package (SAS Inc., Cary, NC, USA). The means were separated using Fisher’s protected least significant difference (LSD, *a* = 0.05), while the *P* values for the *PcTubB* expression analysis were determined using the Student’s *t* test.

**Figure 2:**
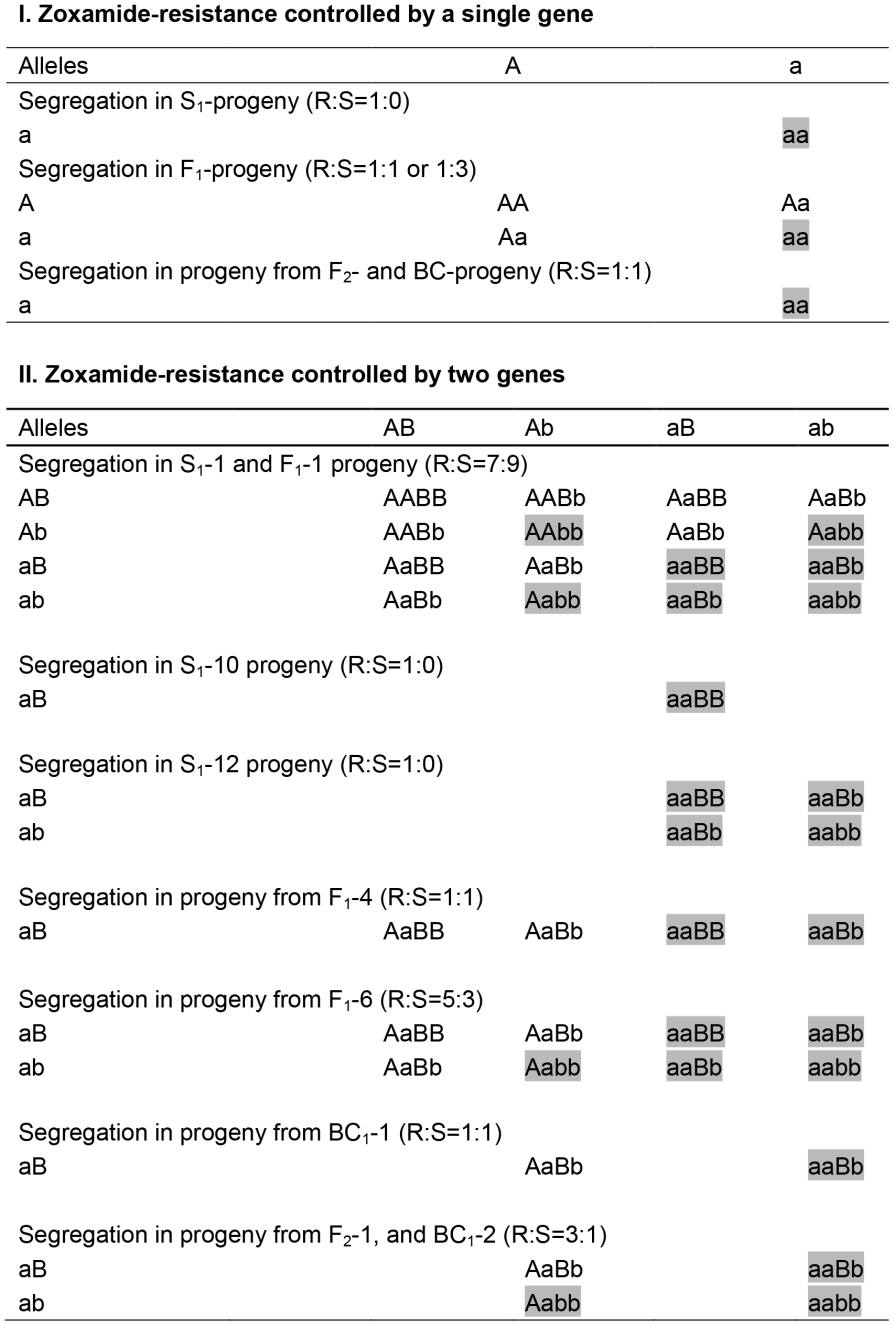
Genotype segregation pattern and expected phenotype of S1, F1, F2 and BC progeny according to the one-gene and two-gene resistance models.

The chi-squared test was used to compare the segregation ratios in the S_1_, F_1_, F_2_, and BC_1_ progeny, which compared the observed ratios of resistant versus sensitive isolates with the predicted values according to both the one- and two-gene resistance models (Figure 2). The one-gene model defines the zoxamide resistance of *P. capsici* as being controlled by a single major gene with incomplete dominance, while the two-gene model, describes the interaction of two genes, where the presence of one or two homozygous recessive genes can cause zoxamide resistance as a result of a complementary effect.

## Results

### Zoxamide-sensitivity of *P. capsici* Isolates

**Figure 3:**
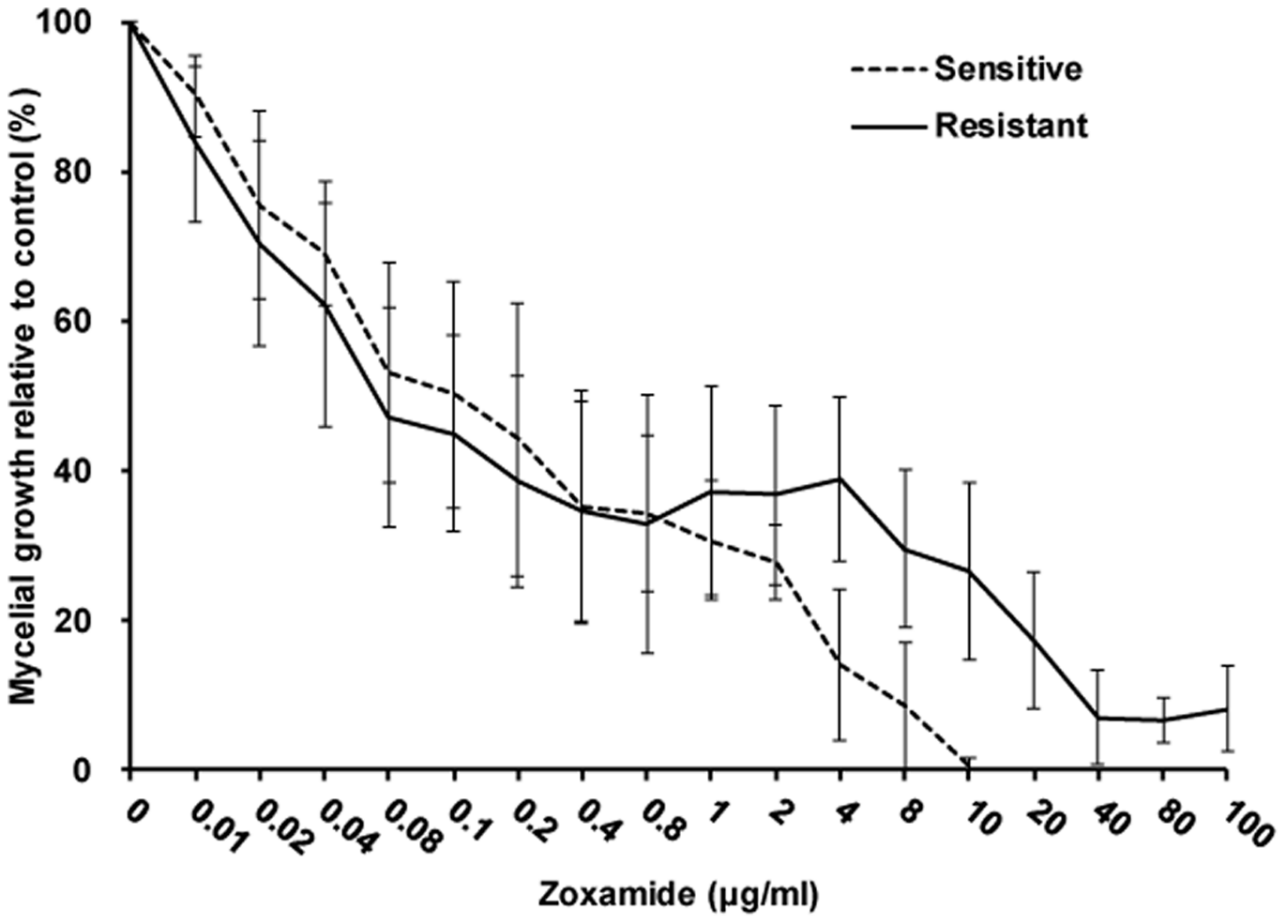
Dose response curve of *P. capsici* isolates grown on zoxamide-amended media. Measurements were made after 4 days of incubation at 25°C.

The *in vitro* fungitoxicity tests using zoxamide-amended media revealed that the sensitive and resistant isolates (Table 1) responded quite differently (Figure 3). The growth rate of the sensitive isolates declined dramatically with increasing concentrations of zoxamide. The inhibitory effect appeared to be directly proportional to the fungicide concentration and the minimum inhibition concentration (MIC) was approximately 10 µg ml^−1^. Interestingly, the resistant isolates responded similarly at the lower concentrations, between 0.01 to 0.8 µg ml^−1^, exhibiting a linear decline in growth, but were less affected at the higher concentrations. This trend was first apparent when the concentration was between 0.8 to 4 µg ml^−1^, but was not statistically significant until the concentration increased to >4 µg ml^−1^. Furthermore, the growth of the resistant isolates was never completely inhibited, even at the highest concentration of 100 µg ml^−1^.

### SNP Investigation of the β-tubulin Gene in *P. capsici*

The full length β-tubulin gene was successfully amplified from the cDNA samples of both the sensitive and resistant isolates, but no difference was found between the *PcTubB* sequence of the two (data not shown). This lack of SNP, particularly the known mutations in the β-tubulin gene conferring zoxamide-resistance in *B. cinerea*, indicates that the mechanism of resistance in *P. capsici* does not correspond to an altered target site.

### Expression Level of β-tubulin Gene in *P. capsici*

The datasets from the RT-PCR were merged as no statistical differences could be detected between the *PcTubB* mRNA levels (*P* = 0.851) from the different biological replicates (Table 2). No significant differences (*P* = 0.587) were found between the relative expression levels of the resistant and sensitive isolates, which ranged from 1.01.5 and 1.01.7, respectively. However, with the exception of isolates PCAS1 and XH38-10, the expression levels of the *PcTubB* gene increased significantly with the addition of zoxamide (*P* = 0.018 and *P* = 0.031; Table 2), although there was significant difference in mRNA levels between the lower concentrations of zoxamide (0.1, 0.5, and 1 µg ml^−1^) and the higher ones (5, 10, and 50 µg ml^−1^) in either the resistant or the sensitive isolates (*P* = 0.410 and *P* = 0.703; Table 2).

**Table 2:** Relative expression of the *TubB* gene in *Phytophthora capsici* isolates.

		Relative expression of *PcTubB* in the presence of zoxamide (µg ml^−1^)^b^						
Isolate	Sensitivity^a^	0	0.1	0.5	1	5	10	50
HX-1	S	1.0±0.0	2.2±0.2	2.6±0.5	2.3±0.2	2.8±0.5	1.9±0.2	2.3±0.4
PCAS2	S	1.7±0.1	1.9±0.2	4.1±0.5	2.4±0.4	3.1±0.3	4.1±0.4	2.8±0.2
PCAS1	S	1.0±0.0	1.1±0.1	1.0±0.1	1.1±0.2	1.1±0.1	0.7±0.1	1.6±0.1
RZ3-5	R	1.2±0.1	2.5±0.3	2.4±0.0	2.2±0.3	1.3±0.0	1.8±0.0	1.4±0.0
RZ13-2	R	1.5±0.1	1.6±0.1	2.1±0.1	2.0±0.3	1.9±0.1	1.6±0.2	2.4±0.2
XH38-10	R	1.1±0.1	1.2±0.2	0.8±0.1	1.2±0.3	0.6±0.1	1.2±0.2	1.5±0.2
XH38-13	R	1.0±0.1	3.1±0.2	3.7±0.6	3.2±0.2	3.5±0.5	2.2±0.8	3.1±0.3

a Isolates were only considered resistant (R) when they were able to grow on PDA plates amended with 30 µg ml^−1^ zoxamide.

b The expression of the *PcTubB* gene was normalized using the *PcWS21* gene and then calibrated to the normalized *PcTubB* mRNA value of the HX-1 isolate in the absence of zoxamide. The mean and standard deviation values indicate the average relative expression between two independent biological experiments. The relative expression was determined in isolates subjected to zoxamide for 6 hours prior to mRNA isolation.

### Collections of Oospore Progeny

A total of 111, 73 and 53 selfed progeny were obtained from the S_1_-1, S_1_-10 and S_1_-12 lines, respectively (Table 3, Figure 1). The number of F_1_ progeny generated by crossing PCAS2 with PCAS1, RZ13-2 or XH38-13 to produce the F_1_-1, F_1_-4 and F_1_-6 lines, respectively totaled 104, 83 and 100 (Table 3, Figure 1). The F_2_-1, BC_1_-1 and BC_1_-2 progeny derived from F_1_-4-15 × F_1_-4-96, F_1_-4-15 × RZ13-2 and F_1_-6-16 × XH38-13, respectively, yielded 82, 98 and 62 progeny, respectively. No oospore progeny were obtained from any of the other crosses (data not shown), and only the F_2_-1 and the two backcross populations (BC_1_-1 and BC_1_-2) were used for the parental and segregation analyses detailed hereafter.

**Table 3:** Segregation of zoxamide-resistance in the sexual progeny of different *Phytophthora capsici* isolates.

			Segregation (Resistant:Sensitive)^a^				
Group	Parental isolate	No. of progeny	Observed	Expected	?*χ^2^*	*P*	Null hypothesis
S_1_-1	PCAS1 (S)	111	53∶58	7∶9	0.7209	0.3959	Accepted
S_1_-10	RZ13-2 (R)	73	73∶0	1∶0	−	−	Accepted
S_1_-12	XH38-13 (R)	53	53∶0	1∶0	−	−	Accepted
F_1_-1	PCAS1×PCAS2 (S×S)	104	47∶57	7∶9	0.0879	0.7668	Accepted
F_1_-4	RZ13-2×PCAS2 (R×S)	83	42∶41	1∶1	0.0120	0.9126	Accepted
F_1_-6	XH38-13×PCAS2 (R×S)	100	56∶44	5∶3	1.8027	0.1794	Accepted
F_2_-1	F_1_-4-15×F_1_-4-96 (R×R)	82	59∶23	3∶1	0.4065	0.5237	Accepted
BC_1-_1	F_1_-4-15×RZ13-2 (R×R)	98	45∶53	1∶1	0.6531	0.4190	Accepted
BC_1-_2	F_1_-6-16×XH38-13 (R×R)	62	45∶17	3∶1	0.1935	0.6600	Accepted

a Sexual progeny were only considered resistant (R) when they were able to grow on PDA plates amended with 30 µg ml^−1^ zoxamide.

### Parentage Analysis of Sexual Progeny

Five isolates: PCAS1, PCAS2, RZ13-2, HX-1, and XH38-13 were screened using eight SSR markers to identify the pattern of sexual hybridization. The zoxamide resistant mutant RZ13-2 was found to contain the same alleles as its parental isolate PCAS1 at all eight loci (Table 4). A similar pattern was also found for the other zoxamide mutant XH38-13, which also shared the same alleles as its parent HX-1. However, both RZ13-2 and XH38-13 contains alleles that were different to those of PCAS2. For RZ13-2, the differences were found in three loci: Pcap3, Pcap4, and Pcap5, while for XH38-13 the differences occurred in Pcap4, Pcap5, and Pcap8 (Table 4). These results indicated that Pcap3, Pcap4, and Pcap5 could be used to characterize the sexual hybridization from the crossing of RZ13-2×PCAS2, while Pcap4, Pcap5, and Pcap8 were suitable for the crossing of XH38-13×PCAS2. Similarly, Pcap3 and Pcap8 were selected for the parentage analysis of the F_1_-4 and F_1_-6 progeny, respectively.

**Table 4:** Genotypic characterization of *Phytophthora capsici* using eight SSR markers.

	Marker^a^							
Isolate	Pcap1	Pcap2	Pcap3	Pcap4	Pcap5	Pcap6	Pcap7	Pcap8
PCAS1	242	263	434	337	285	355	384	412
	242	261	434	321	299	352	381	409
PCAS2	242	266	455	327	290	355	384	433
	240	264	440	309	274	352	384	412
RZ13-2	242	263	434	337	285	355	384	412
	242	261	434	321	299	352	381	409
HX-1	266	294	440	337	299	355	376	375
	250	292	434	334	299	352	372	375
XH38-13	266	294	440	337	299	355	376	375
	250	292	434	334	299	352	372	375
F1-1-10	242	263	440	321	285	355	381	412
	242	261	434	309	274	352	381	409
F1-4-96	242	263	440	321	285	355	381	412
	242	261	434	309	274	352	381	409
F1-6-16	240	260	440	337	290	352	384	433
	240	260	440	327	299	355	384	375

a Allele size for each SSR marker (bp).

All of the single oospore isolates resulting from the crossing tests were subsequently analyzed using the SSR markers mentioned above. Of the 108 sexual progeny from the F_1_-1 population, 104 were determined to be hybrids, and of the 85 F_1_-4 and 103 F_1_-6 progeny 83 and 100, respectively, were found to be hybrids. All of the hybrids identified were used for the subsequent segregation analysis for zoxamide resistance.

### Segregation of Zoxamide-resistance in Sexual Progeny

The results of the chi-squared tests (Table 3) indicated that none of the observed ratios differed significantly from the expected ratios (*P*>0.05). The segregation of zoxamide-sensitivity in the S_1_-1progeny indicated that PCAS1 was heterozygous and that zoxamide-resistance in *P. capsici* was controlled by one or more recessive genes (Table 3). Similarly, the data from the F_1_-1 progeny indicated that PCAS2 was also heterozygous, sharing the same genotype as PCAS1 (Table 3). Furthermore, the segregation ratio of the F_1_-1 progeny suggested that two genes controlled zoxamide resistance in *P. capsici*, and that the genotype of PCAS1 and PCAS2 was therefore ‘AaBb’ (Table 3, Figure 2). The S_1_-10 and S_1_-12 progeny were all resistant to zoxamide, indicating that both RZ13-2 and XH38-13 were homozygous mutants (Table 3). Based on the resistant:sensitive ratios of the F_1_-4 and BC_1_-1 progeny, the genotype of RZ13-2 was likely to be either ‘aaBB’ or ‘AAbb’ (Table 3, Figure 2). The observed ratio of 59∶23 (R:S, expected ratio = 3∶1) in the F_2_-1 progeny (Table 3), inferred that the genotypes of F_1_-4-15 and F_1_-4-96 were ‘Aabb’ or ‘aaBb’, but that the two isolates had a different genotype (Figure 2). Similarly the segregations of zoxamide-resistance in the F_1_-6 and the backcrosses of F_1_-6-16×XH38-13 indicated that the genotypes of XH38-13 and F_1_-6-16 were different and also ‘aaBb’ or ‘Aabb’, respectively (Table 3, Figure 2). Taken together these results demonstrated that zoxamide resistance in *P. capsici* is controlled by two recessive genes, and that isolates showed resistance to zoxamide when either of the two recessive genes were homozygous.

### Biological Characterization of F_1_-progeny

The mating type (A1 or A2) ratio of the F_1_ progeny was approximately 1∶1 (data not shown), and there was only one example of the A1A2 mating type, which was observed in the F_1_-4 progeny (Table 5). The isolates tested differed in their response to temperature with three parental isolates and twenty of the F_1_-progeny being unable to grow at the extreme temperatures: 4°C or 37°C (data not shown). The growth rate of all twenty F_1_-progeny differed from the parental lines with some isolates growing faster while others were slower (Table 5). The production of zoospores differed between isolates with some: F_1_-4-9, F_1_-4-6, F_1_-4-18, and F_1_-4-92, producing significantly more zoospores than their parental isolates RZ13-2 and PCAS2 (*P*<0.05). The zoospore production of the other six isolates from F_1_-4 population showed some degree of variation but did not differ significantly from the parental isolates RZ13-2 and PCAS2 (Table 5). The F_1_-6 progeny also differed in their production of zoospores, with isolates F_1_-6-16, F_1_-6-56, and F_1_-6-88 producing more zoospores than PCAS2, and significantly more than XH38-13 (*P*<0.05), while conversely F_1_-6-18 and F_1_-6-10 produced less than XH38-13, and significantly less than PCAS2 (*P*<0.05). The other five F_1_-6 isolates also showed some degree of variation but did not differ significantly from the parental isolates (Table 5). According to the disease index, the virulence of the twenty isolates also varied, although only one isolate: F_1_-6-5 differed significantly (*P*<0.05), being less virulent than its parent XH38-13. None of the biological characteristics assessed appear to be correlated with zoxamide resistance in any of the 20 isolates tested (Table 5).

**Table 5:** Biological characteristics of F_1_-progeny of *Phytophthora capsici* from sexual crossing.

			Colony diameter (mm)^z^					
Isolate^x^	Sensitivity^y^	Mating type	13°C	20°C	25°C	30°C	Zoosporeproduction (10^5^)^u^	Diseaseindex (%)^u^
**PCAS2**	S	A2	41.5	60.5	62.5	84	2.4	64
**RZ13-2**	R	A1	33.0	56.0	63.0	57.5	0.1	48
**XH38-13**	R	A1	34.0	61.0	67.0	53.5	1.0	92
F_1_-4-6	S	A2	39.0+	58.0+	60.0 −	65.0+	5.0+	76+
F_1_-4-9	R	A1	32.0	53.0	57.0 −	54.5 −	4.3+	100+
F_1_-4-15	R	A2	34.5+	57.0+	62.0	67.5+	1.6	58
F_1_-4-18	R	A1	35.0+	65.5+	67.5+	74.0+	14.3+	78+
F_1_-4-26	S	A1	42.0+	66.5+	75.5+	85.0+	0.3	46
F_1_-4-58	R	A2	31.5 −	54.0	61.5	67.5+	1.1	64
F_1_-4-68	S	A2	42.0+	52.5	61.0	57.5	0.3	90+
F_1_-4-86	S	A2	37.5+	55.0	62.5	68.0+	0.5	86+
F_1_-4-92	R	A1A2	46.0+	67.5+	72.5+	84.0+	10.8+	88+
F_1_-4-96	R	A1	34.0	64.5+	73.5+	61.0+	0.4	72+
F_1_-6-5	R	A2	34.0	65.0+	70.0+	82.5+	1.4	58−
F_1_-6-6	S	A2	43.5+	66.5+	71.0+	77.5+	2.5	78
F_1_-6-10	R	A2	32.0−	61.0	66.0	80.0+	0.4−	86
F_1_-6-16	R	A1	37.5+	50.5−	60.5−	64.5+	3.5+	86
F_1_-6-18	R	A2	33.5	62.0	62.5−	83.5+	0.1−	86
F_1_-6-25	S	A1	33.5	61.0	67.0	72.5+	1.1	90
F_1_-6-56	R	A1	33.5	55.5−	59.5−	59.0+	4.0+	84
F_1_-6-68	S	A1	36.0+	58.0−	66.5	77.5+	1.9	84
F_1_-6-81	R	A2	27.0−	51.0−	61.5−	67.5+	0.7	62
F_1_-6-88	S	A1	36.5+	55.5−	62.5−	70.0+	4.0+	86

x Isolates in bold font are the parent lines of the progeny.

y Sexual progeny were only considered resistant (R) when they were able to grow on PDA plates amended with 30 µg ml^-1^ zoxamide.

z Colony diameters were measured after 5 days of incubation in darkness. The mean values of the sexual progeny within each column were compared to the values from their parents;+and – indicate faster or slower growth, respectively, relative to the parent (*α* = 0.05).

u Zoospore production expressed as number of zoospores per square centimeter of culture; disease index determined from the blight severity scale of inoculated pepper plants.

The mean values of the sexual progeny within each column were compared to the values from their parents;+and – indicate more or less, respectively, relative to the parent (*α* = 0.05).

## Discussion

In the current study, the *P. capsici* resistant isolates had similar sensitivity to zoxamide at lower concentrations as the sensitive ones, but were less sensitive at higher concentrations and could not be completely inhibited even at concentrations far in excess of the MIC of the sensitive isolates. It seems that resistance could be observed just at high rates of zoxmide. Generally, fungicide-resistance is associated with dramatic changes in sensitivity, the resistance level being defined by the EC_50_ value [44], [50]. However, it has been suggested that in the case of *P. capsici*, resistance to zoxamide is better defined according to the EC_90_ value [1]. Nonetheless field isolates developing this type of resistance could still represent a problem for disease control with fungicides.

Zoxamide is an anti-oomycete fungicide that disrupts the microtubule cytoskeleton by binding to β-tubulin [29]. The presence of zoxamide in this study was found to increase the expression of the *PcTubB* gene, reflecting the fact that zoxamide targets the PcTubB protein. Pathogens often develop resistance to the fungicide through alterations to the target protein [35], [38], [39]. However, no difference was found between the mRNA sequences and expression levels of *PcTubB* of the zoxamide-resistant and sensitive isolates, indicating that the resistance did not arises from changes to the target site or from the transcription level. This suggests that *P. capsici* exhibits a novel non-target-site-based resistance to zoxamide. Other groups of anti-tubulin fungicides (BZs and NPCs) are well documented as having serious resistance problem (www.frac.info), and many investigations have shown that resistance to BZs and NPCs is usually conferred by amino acid substitutions in the β-tubulin protein [35]. Investigations of the cross-resistance between zoxamide, carbendazim and diethofencarb have proved a useful approach to understanding the mechanism of resistance to zoxamide [32], [41], [42], and have shown that mutations in their β-tubulin genes can affect their sensitivity to zoxamide. For example, mutation E198A made the *B. cinerea* and *C. gloeosporioides* hypersensitive to zoxamide, while E198K conferred strong resistance [32], [42]. Furthermore, mutations in residues 168, 185, 187, 195, 207, and 240 were found to cause moderate resistance to zoxamide in *B. cinerea* [32]. However, one isolate of *B. cinerea* found to be moderately resistant had no such mutation in its β-tubulin gene, suggesting that alternative mechanisms can also confer resistance to zoxamide [32]. The observations from this study, that zoxamide-resistant isolates of *P. capsici* have no mutations or altered expression of their β-tubulin, indicates that *P. capsici* has developed a similar non-target-site-based resistance to zoxamide. Several non-target-site-based mechanisms have been described for fungicide resistance, including those associated with ABC and MFS transporters, and the metabolic breakdown of fungicides [35], [51]. However, the absence of cross-resistance between zoxamide and other anti-oomycetes fungicides in *P. capsici* [1] indicates that there is no multi-drug resistance, which is usually associated with the resistance conferred by ABC and MFS transporters [52]–[54].

Anti-tubulin compounds have also been developed as herbicides that induce mitotic arrest in plant cells [55]. Several reports about the benzamide herbicide propyzamide (pronamide), structurally similar to zoxamide, have indicated that microtubule-associated proteins and a mitogen-activated protein kinase phosphatase-like gene conferred resistance or hypersensitive to propyzamide in laboratory studies of arabidopsis and tobacco [56]–[58]. These results suggest that proteins involved in microtubule functions could also be responsible for benzamide-sensitivity in *P. capsici*. Indeed, the dose-response curves for the resistant isolates in the current study imply that non-target genes could be responsible for the observed resistance. We hypothesized that non-target genes might be induced at high rates of zoxamide, which contribute to the zoxamide-resistance in *P. capsici*. However, the limited data available regarding microtubule-associated proteins (MAPs) and other related proteins in *P. capsici* unfortunately limits further investigation of this resistance mechanism.

In this study, a total of 18 crossing and 12 selfing experiments were conducted to analyze the segregation ratio of zoxamide-resistance. However, only 3 of the selfings and 6 of the crosses were successful, which perhaps could be accounted for by a lack of oospores production or a low rate of germination as noted in a previous study [59]. Furthermore, it is inevitable that selfed progeny are produced in crossing experiments, for this reason the SSR and SNP analyses proved to be useful tools to detect such selfed progeny in the crossed populations. It has previously been noted that the selection of suitable molecular markers is important for the success of parentage analysis [60]. This study is the first example of SNP being used to analyze the sexual progeny of oomycete fungi, which are remarkable among fungi for their diploid vegetative stage [61].

The gene recombination resulting from sexual reproduction is likely to increase the possibility of resistance developing [28]. Therefore, inheritance of fungicide resistance in oomycete pathogens should be considered an essential factor to risk assessments, especially since it has been noted that the inheritance of single dominant or semidominant gene has been responsible for the rapid development of resistance to phenylamide fungicides in *P. infestans* [19]. The current study suggests that the inheritance of zoxamide-resistance in *P. capsici* is controlled by two recessive genes, and that an isolate exhibits resistance when at least one pair of these alleles is homozygous. However, resistance governed by recessive genes tends to evolve more slowly than by dominant alleles, which produce a resistant phenotype regardless of homozygosity or heterozygosity. Consequently, the risk of *P. capsici* developing zoxamide-resistance in the field is probably quite low, confirming the hypothesis suggested previously that the diploid nature of *P. capsici* and recessive phenotypes represent a low risk of fungicide resistance developing [1], [33].

The current study confirmed that *P. capsici* can develop resistance to zoxamide via the sexual reproduction of two sensitive isolates. A similar report has also shown that selfing of sensitive *P. capsici* isolates, and mass selection can result in the development of resistance to CAA fungicides [28]. These results imply that sexual reproduction is a significant risk for the development of fungicide resistance in *P. capsici*. However, the fitness of fungicide-resistant isolates also plays an important role in the development of resistance once it emerges [62], [63]. In this study, the sexual progeny seemed to be as fit as their parental isolates with regard to their sensitivity to temperature, zoospore production and virulence in host plants, and there was no negative correlation between the fitness of the sexual progeny and the resistance phenotype. These results suggest that sexual reproduction and sensitivity to fungicides in *P. capsici* field population should be monitored to ensure the successful management of diseases caused by *P. capsici*, particularly with regard to the mode of resistance described in the current study.

In summary, we report the mechanism of resistance to zoxamide in *P. capsici*. It is clear from this work that *P. capsici* could develop a non-target-site-based resistance to zoxamide. Furthermore, analysis of the segregation ratio of zoxamide-resistance in the sexual progeny suggests that the resistance to zoxamide is controlled by two recessive genes, and that resistance to zoxamide occurred when at least one pair of these alleles was homozygous. The results imply that the risk of zoxamide-resistance in *P. capsici* is low to moderate, which will be a useful reference for the management of diseases caused by *P. capsici*, especially if two compatible mating types co-exist in the same field.
